# Dual-mode OCT/fluorescence system for monitoring the morphology and metabolism of laser-printed 3D full-thickness skin equivalents

**DOI:** 10.1364/BOE.510610

**Published:** 2024-10-10

**Authors:** Arooj Khalid, Viktor Dremin, Ayman El-Tamer, Maria Surnina, Celine Lancelot, Edik Rafailov, Sergei Sokolovski

**Affiliations:** 1 AIPT, College of Engineering and Physical Sciences, Aston University, B4 7 PH Birmingham, UK; 2 Laser nanoFab GmbH, D-30419 Hannover, Germany; 3 StratiCELL Ltd., Science Park Crealys, 5032 Les Isnes, Gembloux, Belgium

## Abstract

The 3D structure of native human skin is fundamental for studying skin health, diseases, wound healing, and for testing the safety of skin care products, as well as personalized treatments for skin conditions. Tissue regeneration, driven by tissue engineering, often involves creating full-thickness skin equivalents (FSE), which are widely used for developing both healthy and diseased skin models. In this study, we utilized human skin cell lines to create FSE. We designed high-resolution 3D scaffolds to support the growth and maturation of these skin models. Additionally, we developed and validated a cost-effective, custom-built system combining fluorescence spectroscopy (FS) and optical coherence tomography (OCT) for non-destructive analysis of the metabolism and morphology of 3D FSEs. This system proved highly sensitive in detecting fluorescence from key metabolic co-enzymes (NADH and FAD) in solutions and cell suspensions, while OCT provided adequate resolution to observe the morphology of FSEs. As a result, both the 3D FSE model and the dual-mode optical system hold significant potential for use in 3D bioprinting of biological tissues, as well as in the development of cosmetics, drugs, and in monitoring their maturation over time.

## 1. Introduction

The reconstruction of human non- or diseased organs is extremely limited in modern cell molecular biology. It is now possible to fabricate large and complex multi-layered tissues like skin, cardiac tissue, bone tissue, liver, lung, etc. in health or pathology for research and clinical applications [1]. For decades, two-dimensional cell cultures were used in cell biology research as the gold standard. Studying cellular responses in 2D cell culture models is valuable but falls short when exploring the complex interactions that occur in 3D tissues and organs with their biochemical and biomechanical microenvironments. To address this, 3D tissue models provide a more realistic platform, playing an increasingly important role in drug and cosmetics development and testing [2–4].

3D bioprinting represents a cutting-edge approach to generating artificial 3D tissues, employing a variety of cell types, growth factors, and components of the extracellular matrix (ECM) [1], along with the fabrication of 3D scaffolds [5]. Microporous 3D scaffolds, designed to emulate the ECM, offer an enhanced microenvironment that supports cell migration, proliferation, and the overall growth of tissue. A multitude of techniques are available for this purpose, including extrusion, laser-induced forward transfer (LIFT), DLP-based 3D printing, and electrospinning [6]. Some of these methods utilize hydrogel-based bioinks and photopolymers to accommodate cells *in vitro* and foster the development of tissues with specific functions. Particularly, 3D polymeric scaffolds serve as a vital tool in this context. The application of two-photon polymerization (2PP) [7] allows for the precise structural design of 3D scaffolds, achieving high resolution and complex structures suitable for nanoscale applications. Such 3D scaffolds are instrumental in creating tissue constructs for the study of various tissue types, including muscle, bone, cornea, and skin [8].

For fundamental research, drug testing, and clinical studies, human full-thickness skin equivalents (FSEs) have been pronounced as a close substitute for the true skin. FSE can be constructed by using skin cells primarily or TRET-immortalized keratinocytes and fibroblasts [9]. For this purpose, cultured cells are utilized for creating and generating 3D human organs for the purpose of replacement and grafting [10]. To obtain adequate information about 2D cell cultures and 3D tissues, optical imaging and spectroscopies are valuable tools for the estimation of morphology and biochemical details [11,12]. A broad range of fluorophores and chromophores exist in the skin tissue, such as lipofuscin, collagen, elastin, porphyrins, protein, haemoglobin, NADH, and FAD. Fluorescence of two of the most important endogenous co-enzymes in mitochondrial metabolism and fluorophores, reduced nicotinamide adenine dinucleotide (NADH) and flavin adenine dinucleotide (FAD), can act as a biomarker of the metabolic activity of cells and tissues [13–18]. These two coenzymes are significant markers of cellular aging and maturation [19], and they are also found in the keratinocytes and fibroblasts of the FSE. Besides fluorescent spectroscopy used for assessment of the cellular metabolic activity, OCT imaging can non-disruptively show the 3D FSE morphology and provide a non-invasive real-time cross section of FSE with an ideal balance between penetration depth of 2 mm and resolution of 10-15 µm [20].

Our goal was to create biocompatible 3D scaffolds and develop a dual-channel system combining OCT and FS. This system is designed to enable both morphological and metabolic evaluation of the 3D-engineered FSE.

## 2. Materials and methods

### 2.1 Preparation of NADH and FAD chemical solutions

10 mM stock solutions of NADH and FAD (Sigma-Aldrich, USA) have been prepared in Dulbecco’s phosphate-buffered saline (DPBS, Sigma Aldrich). Eight different concentrations of 1 mM, 0.6 mM, 0.3 mM, 0.1 mM, 0.03 mM, 0.01 mM, 0.003 mM, and 0.001 mM with have been prepared from the initial stock solution and kept in -20 ^o^C. The stock solution was shielded in metal foil for prevention the effect of photobleaching due to natural room light. For all measurements of NADH and FAD, a quartz cuvette is used having a transmission optical path of 10 mm.

### 2.2 Human primary fibroblasts and HaCaT cell culturing

The stock of primary human fibroblasts and immortalized keratinocytes (HaCaT cells) were provided by StratiCELL (Belgium). Primary human fibroblasts were grown in Dulbecco’s Modified Eagle’s Medium (DMEM, Thermo Fischer Scientific, UK) media with 10% of Foetal Bovine Serum (FBS, Sigma-Aldrich, UK) and 1% Penicillin/Streptomycin (Thermo Fischer Scientific, UK). Cells were seeded in a T75 flask (Corning, USA), incubated at 37 ^o^C temperature humified with 5% CO_2_. Cells were seeded at 4.5 × 10^5^ cells/T75. After 5 days of culturing, when cells reached 90% of confluence, Trypsin-EDTA solution (Sigma-Aldrich, USA) was added to the flask and incubated for 2 to 3 minutes at 37 ^o^C. After trypsinization, cells were centrifuged (at 800 RPM for 5 minutes) and were utilized for experimental purposes, and the remaining cells were stored in FBS with 10% dimethyl sulfoxide (DMSO) (VWR Chemicals BD) and stored at -80 ^o^C for further use.

HaCaT cells were grown in Dulbecco’s Modified Eagle’s Medium (DMEM) high glucose media (Sigma-Aldrich, USA) with 10% fetal bovine serum and were seeded as described above.

### 2.3 Human primary fibroblasts and HaCaT cell culturing

The viability of the cells is estimated using an improved Neubauer chamber with Trypan Blue (Stemcell Technologies Inc.) 0.4% [21]. The viable and non-viable cells of the primary fibroblasts and HaCaT cells are identified and counted using an improved Neubauer chamber and inverted microscope (Motic AE 2000, Motic, UK). For fibroblasts and HaCaT cells, the viability of all cell lines cultured was 80 ± 10%.

### 2.4 Laser-printed 3D scaffolded FSE

Two-photon laser-printed 3D scaffolds fabricated from biocompatible commercially available material (Dental-LT-Clear) (Formlabs, Germany). 2PP with BioScaffolder system (Laser nanoFab GmbH, Germany) was used to produce 9 mm round scaffolds. The architecture of the 3D scaffold was comprised of two layers with 80 µm height, 30 µm cylinder wall thickness and an inner pore diameter of 240 µm [22]. Scaffolds were sterilized using 70% ethanol and UV light for 30 min. Polystyrene clear flat bottom TC-treated 6-well plate (Corning, Falcon, USA) was used for cell seeding.

PEGylated fibrinogen was prepared composed of PEG-NHS (Sigma-Aldrich, USA) and fibrinogen (Sigma-Aldrich, USA). Human primary fibroblasts (Fb) of 1 × 10^5^ per ml were suspended in PEGylated fibrinogen solution (FG) along with 100 µl CaCl_2_ (50 Mm) and 100 µl thrombin (40 IU/ml in TBS) (Sigma-Aldrich). This composition was added into the scaffold inside the 6-well plate (see below). The cell culture plate was placed in the incubator for 1 hour for polymerization. Afterwards, 4 ml of medium (DMEM + 10% FBS + 1% Penicillin/Streptomycin) was added to the wells and was kept at CO_2_ incubator at 37 ^o^C for 48 h. The HaCaT cells of 9 × 10^5^ cells/ml were seeded on the top of the fibrinogen gel. The completed sets of FSEs on the scaffolds in 6-well plates were cultivated with media 4 ml of DMEM + 10% FBS with 10 mg/ml aprotinin changed every 2 days, for the final maturation of FSEs.

### 2.5 Statistical analysis

The results of experiments were averaged and presented as mean ± standard deviation (SD). Data and statistical analysis were performed using OriginPro software (OriginLab Corp., USA). Taking into account the small sample sizes, nonparametric Mann–Whitney U-test were used to confirm the reliability of differences in the results. Values of p < 0.05 were considered significant.

### 2.6 Histological analysis of 3D FSE with haematoxylin and eosin staining (H&E)

The FSE, along with the scaffold, was taken from the scaffold holder and fixed in 4% formaldehyde at room temperature and embedded in paraffin using the RHS-2 microwave. Haematoxylin and eosin (H&E) were used for staining 6 µm sections of skin tissues using microtome [23]. The sections are then dehydrated in an ethanol bath, isopropanol bath and Ultraclean bath. Finally, the FSE sections were mounted by using an Ultra kit.

## 3. Results

### 3.1 Development of a combined OCT and fluorescence spectroscopy system

The FSE approach in skin molecular biology was used to study human skin development in dynamics where H&E histology is the main visualisation technique of FSE morphology. When preparing samples for H&E histology, the process irreversibly damages the FSE, rendering the method both costly and labour-intensive. Why we have chosen OCT imaging and FS for non-destructive optical interrogation of 3D FSE morphology and metabolic activity.

**Fig. 1.**
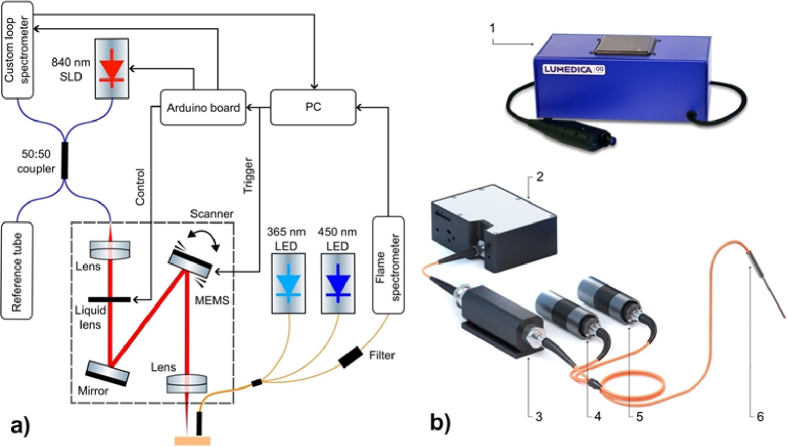
(a) General scheme of the experimental setup and (b) overview of channels: 1 – Lumedica OCT imaging system, 2 – spectrometer, 3 – filter holder with long-pass filters, 4 – LED 365 nm, 5 – LED 455 nm, 6 – optical probe.

Therefore, we developed the experimental setup, including the FS system with OCT (Fig. 1). The FS system with fibre optical probe containing emitting and collecting fibres was developed for recording the fluorescence intensity (FI) spectrum after 365 and 455 nm excitation. The FI was measured based on autofluorescence of the biomarkers exited with 365 and 455 nm LED emitters. Therefore, FS system doesn’t require dye neither for NADH nor for FAD identification.

The choice of emitter wavelengths was based on NADH and FAD fluorescence excitation features [24–27]. To reduce the photobleaching effect, the radiation power of the 365 nm LED excitation source (M365FP1, Thorlabs, USA) did not exceed 0.35 mW. The output power of the 455 nm LED excitation source (M455F3, Thorlabs, USA) was even lower – 0.1 mW. Fluorescence radiation in the range of 350-1000 nm was analysed using a FLAME-T-VIS-NIR-ES spectrometer (Ocean Insight, USA). To attenuate the backscattered radiations of the LED sources, Ø12.5 mm long-pass filters of 400 nm for 365 nm excitation LED and 500 nm for 455 nm excitation LED (Edmund Optics, USA) were used. Fiber-coupled INLINE-SFH accessory (Ocean Insight, USA) was used as a filter holder. The R400-7-UV-VIS optical probe (Ocean Insight, USA) has 6 illumination fibres around 1 detection fibre. All fibres have a 400 µm core size. The numerical aperture of the fibres is 0.22.

For non-invasive visualization of morphological features of FSEs, the compact, reliable, and easy-to-use Lumedica OCT imaging system was selected [28]. The light source is provided by a super luminescent diode with a centre wavelength of 840 nm, that is coupled with an interferometer and a high-resolution spectrometer. The 840 nm laser in the OCT imaging system was used to scan artificial FSE, which primarily consists of fibrinogen gel and cell layers. These materials are fully transparent at this wavelength, unlike real skin. A scanner is also mounted with the system for scanning samples. Resolution depth is 5 microns in tissue, transverse resolution 15 microns, and linear scan range 7 mm. These parameters are influenced by the choice of this specific system.

### 3.2 Assessing FS module sensitivity to the NADH and FAD concentration range

**Fig. 2.**
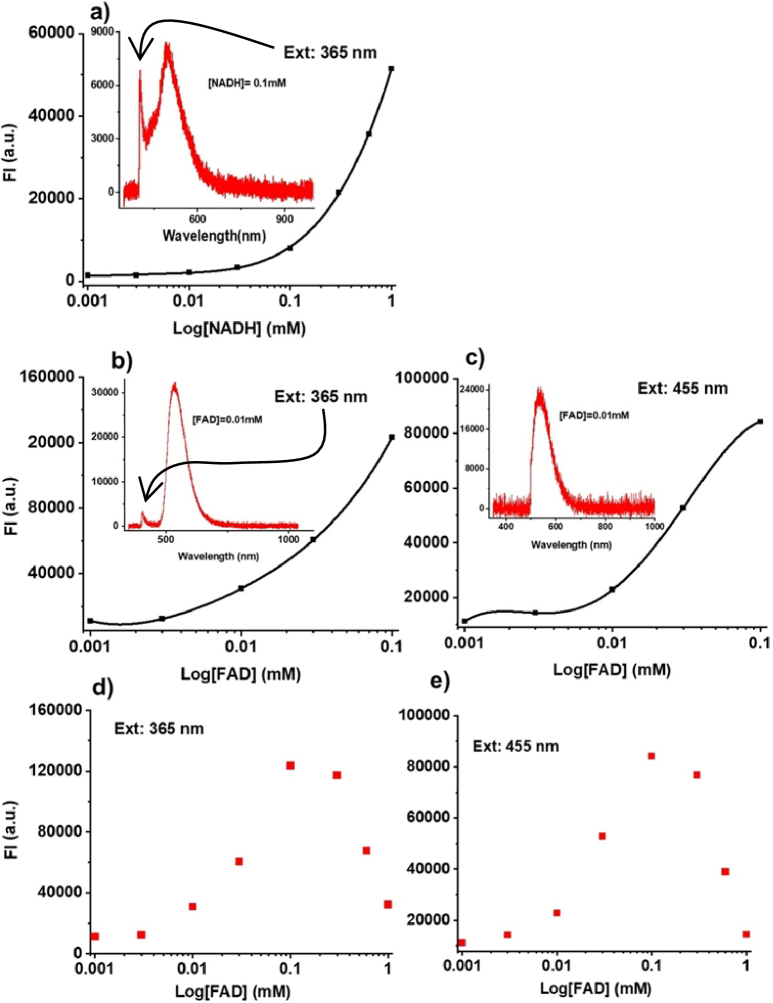
Fluorescence intensity measured in NADH (a) and FAD (b-e) solutions of different concentration at 365 nm excitation with 400 nm long-pass filters (a,b,d) and 455 nm excitation with 500 nm long-pass filters (c,e) with custom-built FS module. Insertions: representative fluorescence spectra of NADH (0.1 mM) and FAD (0.01 mM) for different excitation wavelengths. Each measurement was repeated three times (n = 3). Experimental data from a, b, and c were fitted with a 4-order polynomial function utilising the OriginPro program. (d, e) Quenching effect in FAD solutions.

The sensitivity of the built-in FS module to the concentration of NADH and FAD was evaluated on solutions of the corresponding chemicals in DPBS. The fluorescence spectra of NADH solution at 365 nm and FAD solution at 365 and 455 nm and their fluorescence intensity (FI) at different concentrations are shown in Fig. 2. The representative NADH and FAD fluorescence spectra demonstrate partially cut excitation after 365 nm peak with 400 nm dichroic long-pass filter (Fig. 2(a),(b)) and none of the 455 nm excitation peak is left after the 500 nm filter. FI values demonstrated a strong correlation with the concentration of NADH and FAD in the solution – the higher FI value corresponds to the coenzyme’s higher concentration (Fig. 2(a)-(c)).

Data shown in Fig. 2(a) represents NADH FI excited with 365 nm. Even at the highest 1 mM NADH concentration, FI saturation wasn’t indicated. Figure 2(b),(c) represents the FAD excitation at 365 nm and 455 nm, respectively. At FAD concentrations above 0.1 mM, the concentration quenching effect is observed (Fig. 2(d),(e)). This effect is due to the absorbance of the excitation light by the sample itself [29]. These experimental results confirmed that the FS module demonstrated sensitivity thresholds for both main metabolic coenzymes within their nominal values as they were measured in the mouse spleen tissue for NADH in the range of 90-520 µM and for FAD of 80-400 µM [30].

After the validation of FS mode sensitivity with different concentrations of NADH and FAD, we validated it using HaCaT cell and fibroblast suspensions.

### 3.3 NADH and FAD levels in HaCaT cell and fibroblast suspensions

**Fig. 3.**
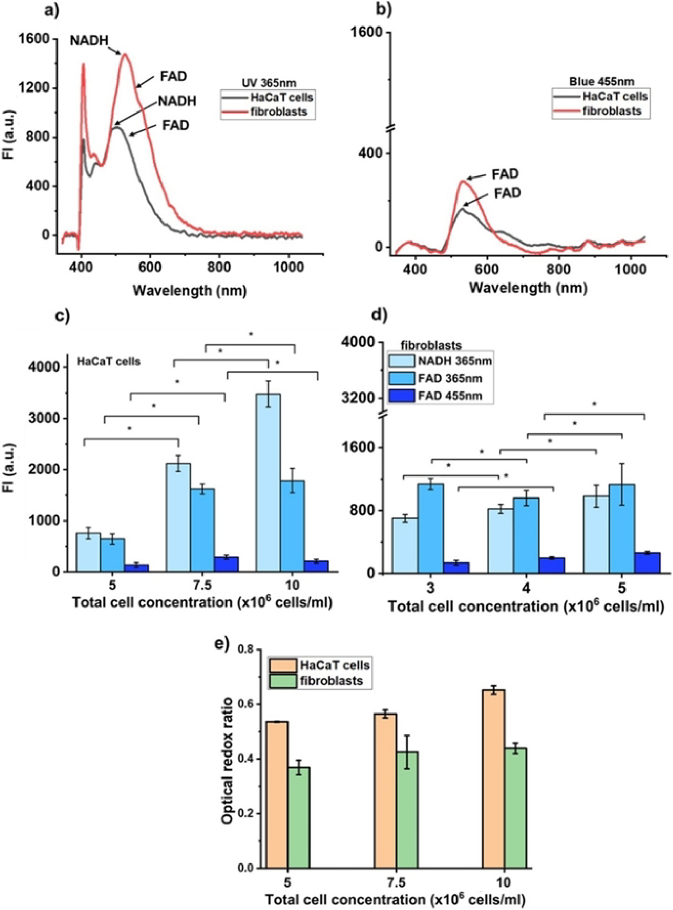
FS selectivity and sensitivity evaluation in HaCaT cell and fibroblast suspension at a concentration of 5 × 10^6^ cells/ml for both excitation wavelengths: (a) UV 365 nm and (b) blue 455 nm. NADH and FAD FI were measured at their maxima (492 nm and 535 nm correspondently) of cell spectra. (c) FI measured in HaCaT cells and (d) fibroblasts at three different concentrations (n = 3, * confirmed statistically significant differences between different cell concentrations, *p *< 0.05); (e) comparison of ORR of HaCaT cells and fibroblasts after exposure at 365 nm at their correspondent concentration (c,d). Data are presented as mean ± SD.

The selectivity and sensitivity of the FS module in detecting NAHD and FAD contents in living cells were also evaluated on immortalized keratinocyte (HaCaT) and human primary fibroblast suspensions with the background fluorescence from other cellular endogenous chromophores (Fig. 3).

Figure 3(a),(b) represents fluorescence spectra for both cell types and wavelength excitations. FI of NADH and FAD was estimated at the maximum of the fluorescence spectrum. The cell solutions were resuspended in measuring cuvette before fluorescence measurements to guarantee uniform cell saturation across the volume. The cell uniformity per say was the same level because cells harvested for all experiments were at the confluency around 80% for both cell types.

As can be seen, the FI of NADH is significantly higher than the FI of FAD. Figure 3(c),(d) represent maximum FI related to the metabolic activities of viable cells at three different concentrations: 5 × 10^6^, 7.5 × 10^6^ and 10 × 10^6^ cells/ml. It should be noted that other cellular fluorophores can increase actual FI^NADH^ and FI^FAD^ values [31]. Since FI values of NADH and FAD were compared in different cell concentrations, the additive effect of other cellular fluorophores can be ignored.

For NADH, with increasing cell concentration the FI also increases. When FAD was excited at 455 nm, the highest FI was detected at a concentration of 7.5 × 10^6^ cells/ml, a further increase in cell concentration did not reduce FI^FAD^.

For label-free detection of changes in cell and tissue metabolism type [32], optical redox ratio (ORR) is often calculated. The change in ORR usually indicates relative changes in oxidation phosphorylation in mitochondria [33]. Cell or living tissue ORR can potentially demonstrate the integrity of the cellular energetic metabolism and, in turn, indicate the state of the cell/tissue development/maturation. There are many ways how to calculate ORR [34]. In our study, the ORR is calculated using the following formula ORR = FI^NADH^/(FI^NADH^ + FI^FAD^). The use of FI^NADH^ and FI^FAD^ in the calculation of ORR is associated with changes in glycolytic and oxidative phosphorylation metabolism [35]. If ORR increased, it indicates that metabolism had shifted to the glycolytic type due to the prevalent decrease of FAD generation or the increase in NADH concentration due to glycolysis. Contra versa, an increase in oxidative phosphorylation reduces NADH while FAD increases [34].

Figure 3(e) shows the ORR of HaCaT cells and fibroblasts after UV exposure. It is revealed that the change in ORR of both cell types is not significantly different. The changes of FI^NADH^ and FI^FAD^ at UV exposure led to an increase in ORR for both cell types.

### 3.4 NADH and FAD levels in HaCaT cell and fibroblast suspensions

**Fig. 4.**
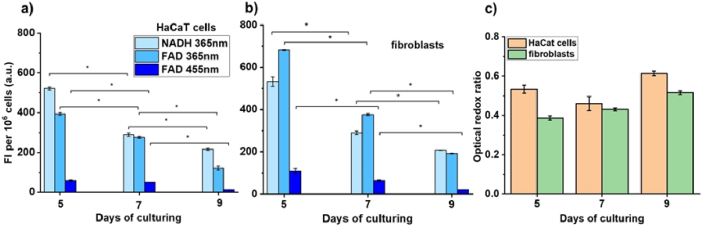
The FI of (a) HaCaT cell and (b) fibroblast suspensions at the different culturing intervals. (c) Comparison between cellular ORR of HaCaT cells and fibroblasts after exposure at 365 nm for different days of culturing. Data are presented as mean ± SD (n = 3, * p < 0.05).

Furthermore, HaCaT cells and fibroblasts that were cultured over various durations (5, 7, and 9 days) underwent evaluation. Figure 4(a),(b), correspondingly, represent the dependence of FI of the main coenzymes of mitochondria in HaCaT cells and fibroblasts for 5, 7 and 9 days. The shift of FI for both NADH and FAD towards their decrease was prominent for both cell types at both excitation wavelengths.

Figure 4(c) showcases a comparison of the oxygen reduction reaction (ORR) for two types of cells cultured over periods of 5, 7, and 9 days. Despite the increased duration of culturing for both cell types, the ORR levels remained constant, suggesting no significant change in general metabolic activity. This is further supported by a steady decline in the peak fluorescence of coenzymes (as shown in Fig. 4(a) and 4(b)), indicating that the processes of anabolism and catabolism remained stable. Consequently, this stability ensures that glycolysis and oxidative phosphorylation processes are in equilibrium [34,36]. However, the observed progressive reduction in the FI^FAD^ and FI^NADH^ due to the aging of the culture might signal a decrease in overall metabolic activity. This typically occurs when a 2D culture reaches a certain level of confluency or when a 3D model achieves maturation [37].

The FS module has shown that as cells mature, there's a noticeable reduction in NADH, FAD, and overall fluorescence. This decline is attributed to the lower energy requirements needed to sustain elevated cell growth rates. Minor alterations in ORRs indicate that, over extended periods of cultivation, both types of cells started to equally rely on glycolysis and oxidative phosphorylation. This adaptation ensures optimal energy production by utilizing both anaerobic and aerobic metabolic pathways [32].

### 3.5 Culturing FSE on two-photon printed 3D scaffolds with customised architecture

There are some limitations in using the 2D skin cell monolayer cultured on a solid surface under certain physio-chemical conditions, and the 3D skin models undeniably became a valuable approach for studying pathologies and new drugs and cosmetics [38] testing. Therefore, 3D FSE makes possible the co-culturing of separate layers of different cells (fibroblasts, keratinocytes, melanocytes, etc.) to proliferate and interact vertically and horizontally with all cell components within an extracellular matrix developing highly comprised artificial tissue [39]. Human full-thickness skin equivalents have multiple uses and benefits in examining not only how newly developed drugs/cosmetics with epidermal and dermal cells but also epidermal gene expression and understanding the roles of these two layers in disease modelling and wound healing. It is also a promising tool to significantly decrease animal testing [40,41].

**Fig. 5.**
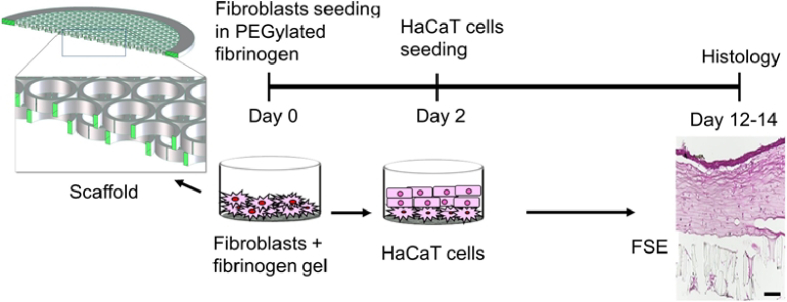
Schematic representation of FSE developing on the 2PP printed 3D scaffolds, including scaffold structure, cell seeding, and FSE histology. Scale bar: 200 µm.

To generate a biomimetic human skin model for more predictive and relevant human skin research, 3D FSE has been developed by using primary human fibroblasts and HaCaT cells on 3D scaffolds printed with a two-photon printing laser system, as shown in Fig. 5.

The mixture of human primary fibroblasts in fibrinogen gel laid on a two-layer scaffold (Fig. 5) was capable to support HaCaT cell layer seeded on the top to develop FSE. The resultant FSE is highly differentiated and closely mimics the architecture of human skin (Fig. 5, histology section) [42]. The 3D polymeric scaffold provides an instructive template for *in vitro* comfort cell placement for tissue development with certain functional properties [22].

Histological analysis of FSE on day 12 denoted the HaCaT cell layer on the fibroblast-fibrinogen gel layer on top of the porous scaffold, supporting the required exchange of nutrients between the medium and FSE. After 12 days of culture, the HaCaT cell-based layer has been successfully developed at the air-liquid interface, effectively mimicking the native morphology of the human epidermis [43].

### 3.6 FS and OCT Imaging of 3D tissues in maturation dynamics

Well-known methods of metabolic and morphological evaluation of tissue and 3D *in vitro* models such as FSE are based on the immunoassay of NADH and FAD or other energy-storing molecules from crude cell or tissue extracts and H&E-stained biopsies. Both approaches are damaging and lead to the full destruction of expensive samples. To avoid this, we developed a dual-mode OCT-FS system allowing contactless monitoring of fibroblasts in fibrinogen gel (Fb + FG) and FSE morphology and metabolism in real-time. We compared the morphology and metabolic activity at 7- and 14-day-old of Fb + FG and FSEs.

**Fig. 6.**
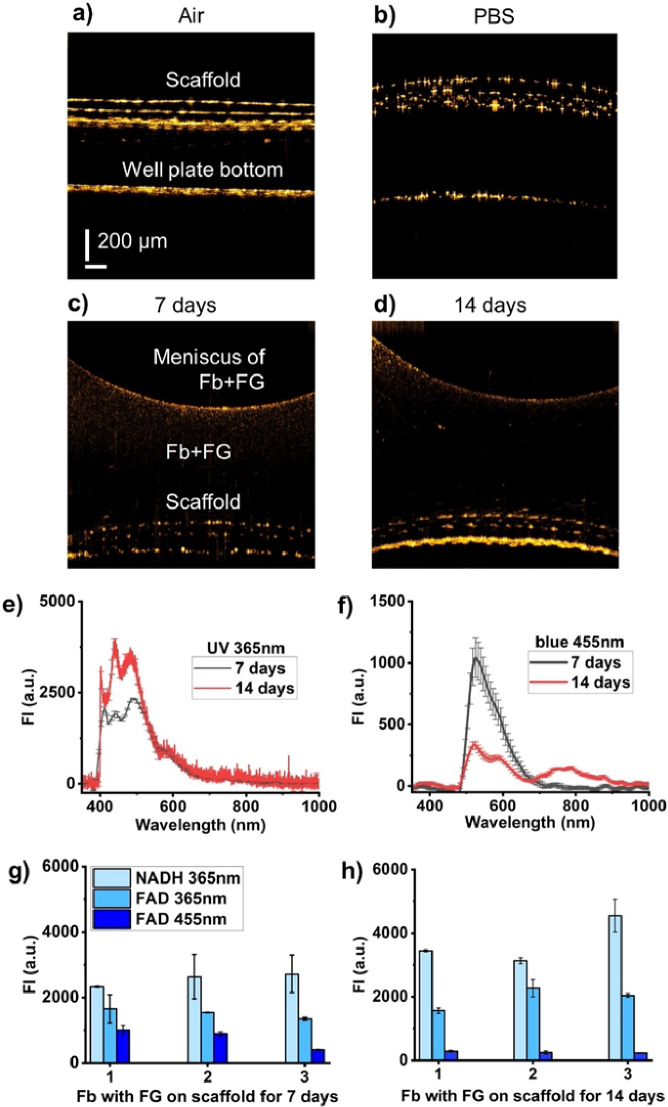
The FS-OCT system evaluated the metabolism and morphology of 3D Fb + FG on scaffolds. Representative OCT image of scaffold in the well plate (a) without and (b) with PBS, (c) 7-days-old Fb + FG and (d) 14-days-old Fb + FG. Scale bar: 200 µm. (e, f) Fluorescence spectra of 7 and 14 days of culturing of Fb + FG at 365 nm and 455 nm excitation, correspondingly. (g, h) Maximum FI of 7 and 14 days of culturing of Fb + FG at different excitation wavelengths. Data are presented as mean ± SD (n = 3, 3D samples).

**Fig. 7.**
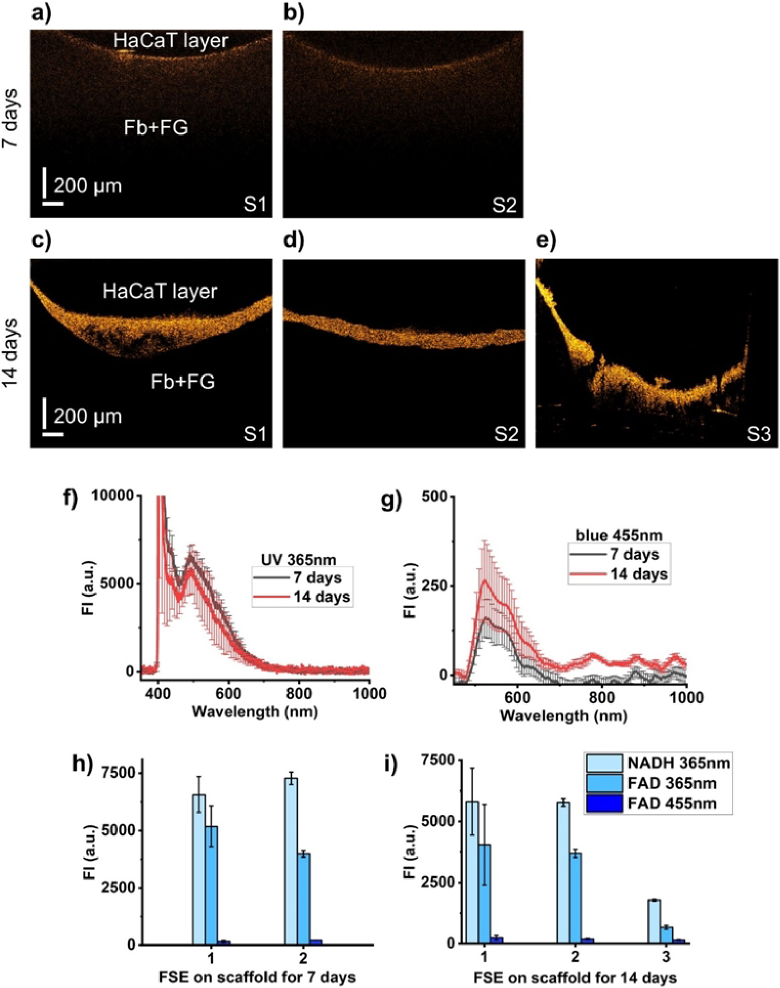
Metabolic and morphological evaluation of completed FSE after 7 and 14 days of culturing. (a, b) Representative OCT images of 7-day-old two FSEs (S1, S2). (c-e) Representative OCT images of 14-day-old three FSEs (S1, S2, S3). Scale bar: 200 µm. (f, g) Fluorescence spectra of 7 and 14 days of culturing of FSE at 365 nm and 455 nm excitation, correspondingly. (h, i) Maximum FI of 7 and 14 days of culturing of FSE at different excitation wavelengths. Data are presented as mean ± SD (n = 3).

OCT images demonstrate certain similarities to FSE histology stained with H&E (see Fig. 6(a)-(c) and 7a-e), proving that OCT has great potential in representing accurate dimensions of the artificial tissues.

First, with the help of OCT, we obtained images of the scaffold in the well plate without and with PBS (Fig. 6(a),(b)). Next, we examined the Fb + FG morphology on days 7 and 14. Figure 6(c),(d) demonstrate the meniscus formation. In general, it can be concluded that Fb + FG is a low-scattering and weakly absorbing medium, which is emphasized by the presence of a scaffold on the image's bottom side. Figure 6(e),(f) also shows NADH and FAD fluorescence spectra measured from 7- and 14-day-old Fb + FG grown on the scaffold. The measurement results show that tissue growth leads to an increase in the level of NADH fluorescence and a decrease in the level of FAD (up to 50-60%) (see also Fig. 6(g),(h)). Based on our modeling data for real skin, it is assumed that the depth of light penetration into printed tissue may differ between 365 nm and 455 nm. The penetration depths for these wavelengths could be 300 µm and 500 µm, respectively [18]. Printed tissues are likely to exhibit deeper penetration while maintaining the same proportional relationship.

Morphological evaluation of fully developed FSE cultured for 7 and 14 days on different scaffolds (S1, S2, S3) showed a noticeable difference in the thickness of the layers of *in vitro*-developed HaCaT cells (see Fig. 7). In the first 7 days of cultivation, the HaCaT cells layer is practically undeveloped (Fig. 7(a),(b)). An additional 7 days of cultivation leads to a significant increase in the thickness of the top layer (Fig. 7(c)-(d)).

The layer of fibroblasts incorporated into the fibrinogen gel is located on the top of the scaffold and provides a base for the growth of HaCaT cells, similar to the epidermis of real skin. The layer of HaCaT cells has been detected through a highly backscattered area of the constructed FSE. According to H&E histology (Fig. 5), the thickness of the HaCaT layer is ∼150 µm. This corresponds to the values of the thickness of the HaCaT layer obtained from OCT measurements. The thickness of the layer developed from HaCaT cells for S1 (Fig. 7(c)) is ∼200 µm. In S1, the diffuseness and some defects of the HaCaT cell layer can be observed. In Fig. 7(d) (S2), the developed FSE model is more stratified and cultured homogeneously as compared to the thickness of cells developed in S1 and S3 (Fig. 7(e)). In S3, raptures and inconsistency of the HaCaT layer are observed. The thickness of the model developed in S2 is ∼90 µm and in S3 – ∼180 µm.

The most remarkable observation was that OCT clearly revealed differences in HaCaT cell layer shapes on individual FSE samples (Fig. 7(a)-(e)), proving that morphology OCT imaging is a powerful approach to evaluating deviations in the development of FSE. The difference between Figs. 6(a) and 6(b) arises from the presence of PBS in sample 6b. The variation between Figs. 6(c) and 6(d) can be attributed to changes in system focus that often happens during OCT imaging. Despite this, all images were processed using consistent intensity thresholds. It is important to note that the post-fixation dehydration process inherent in the H&E staining protocol leads to a reduction in FSE tissues. The thickness of the FSE dermis can decrease by 68% due to the fixation procedure [23]. This contrasts with OCT imaging, which uses fresh, non-dehydrated FSE samples. Thus, our approach provides true information about sample sizes.

**Fig. 8.**
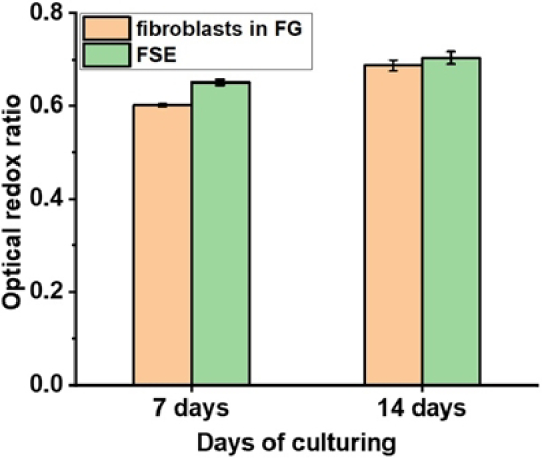
Optical redox ratio of Fb + FG and FSE for 7 and 14 days of culturing. Data is presented as mean ± SD (n = 3).

We also evaluated cellular metabolic activity in FSEs after exposure to UV and blue light. The ORR for both 7- and 14-day-old FSE is not significantly different. Based on NADH and FAD fluorescence measurements (Fig. 7(f)-(i)), it can be seen that the HaCaT cell layer in FSE is metabolically more active compared to the FI of Fb + FG. Insignificant fluctuations in ORR parameters (around 0.6 a.u.) for 7- to 14-day-old Fb + FG compared to FSE (Fig. 8) indicate that glycolysis but not oxidative phosphorylation is dominant for the metabolism of both derma models [34].

Therefore, we think that the most reliable clues about the level of FSE development and maturation can be OCT morphology imaging demonstrating dimensions of the artificial epidermis and dermis layers and changes in the FI of NADH and FAD.

## 4. Discussion

The optical pattern of skin depends upon the interaction of light wavelength with different layers of cells produced by cellular matrixes. OCT imaging can provide real shapes and dimensions of developed artificial skin models and a measurable thickness of the developed epidermis layer formed by the HaCaT cell layer in FSE and reflect its structural defects of the scaffold, Fb + FG, and HaCaT cell layers [23,43]. Since this research fucuses on assessment of the morphology not real human skin but FSEs which mostly consists of fibrinogen gel highly light transferable we used short wavelength OCT system with 840 nm laser.

The fluorescence spectra analysis revealed distinct patterns of FI shifts between UV- and blue-excited emissions across the Fb + FG and FSE models. Specifically, the Fb + FG model demonstrated a notable sensitivity to UV excitation, which we attribute to its relatively simplistic structure and the inherent properties of the fibrin gel matrix. In contrast, the FSE model, which closely mimics the full thickness and complexity of human skin, exhibited a pronounced response to blue light excitation. These findings suggest that the structural composition and thickness of these two models significantly influence their metabolic fluorescence signatures. The FI shifts offer insights into the metabolic dynamics and biochemical composition of the models, reflecting their physiological relevance and potential applications in dermatological research. In the present study, it is assumed that HaCaT cells, fibrin-based dermal matrix and primary fibroblasts absorb incoming incident light. Since the penetration depth for UV and blue is about 200 µm and 400 µm respectively [44], and the thickness of developed FSE is about 2 mm, it can be considered that fluorescence spectra are combined spectra from fibrinogen gel with fibroblasts and HaCaT cells. Our results show that FAD decreases with increasing the culturing time of fibrinogen gel while NADH increases. For FSE, a low concentration of FAD was detected as compared to NADH. The maximum fluorescence intensity of the S2 scaffold (FI^S2^, Fig. 7 g) has the lowest SD values compared to FI^S1^ and FI^S3^, where representative OCT images (Fig. 7(a)-(c)) demonstrated high inconsistency and even raptures (S3) of HaCaT layers. HaCaT layer developed on scaffolds S1, S2, and S3 has distinguishingly different OCT shapes, and their corresponding FI values are also different. Therefore, the variation of FI^S1^ is more prominent than that of S2 and S3. This indicates that our OCT-FS system is sensitive enough to detect the peculiarity in the morphology and corresponding changes in metabolic activities of the artificially developed 3D scaffold FSE.

This optical characterization is not only helpful for the detection of healthy and unhealthy developed skin but also is a non-destructive and reliable method for metabolic assessment of 3D tissue models. For evaluation of our developed OCT system, OCT images of *in vitro* developed FSE are compared with H&E-stained histology along with developed FSE in a 3D scaffold. The dimensions of the fully developed FSE can trustfully be evaluated with OCT since its OCT image demonstrates a high similarity to the histological image of H&E-stained FSE.

This study has demonstrated the validity of our customized FS device combined with OCT imaging for non-damaging assessment of human HaCaT cells and fibroblasts metabolic maturation and morphology of FSE model grown on architecturally customised laser-printed 3D scaffolds. OCT also provides morphological images of the development of the fibroblasts within fibrinogen gel when growing on 3D scaffolds. Therefore, the dual-mode optical system can be considered highly verifying and sensitive and can be used in the future for a full-profile assessment of the maturation and viability of 3D *in vitro* developed models of biological tissues. Second, the OCT module used for contactless evaluation of dimensions and morphological patterns of developing and developed FSEs demonstrated (i) high alikeness to H&E histology patterns, (ii) real skin dimensions, (iii) with the advantage of keeping growing FSEs intact from the first day of culturing to its full maturation. Thus, the dual mode FS/OCT system is an excellent way to study and monitor the metabolism and morphology of developing a 3D scaffolded tissue model *in vitro*.

## Data Availability

Data underlying the results presented in this paper are not publicly available at this time but may be obtained from the authors upon reasonable request.
